# The council of expediency: crisis and statecraft in Iran and beyond

**DOI:** 10.1080/00263206.2019.1585346

**Published:** 2019-04-27

**Authors:** Maziyar Ghiabi

**Affiliations:** Wadham College, University of Oxford, Oxford, UK

**Keywords:** Iran, statecraft, state formation, crisis management, expediency Council, Agamben, *maslahat*

## Abstract

Giorgio Agamben argues that in contemporary governance the use of ‘emergency’ is no longer provisional, but ‘constitutes a permanent technology of government’ and has produced the extrajudicial notion of crisis. The engendering of ‘zones of indistinction’ between the law and its practice is what Agamben defines as a ‘state of exception’. This article adopts the notion enunciated by Agamben and revisits it in the Islamic Republic of Iran. There, the category of crisis has been given, firstly, a juridical status through the institution of *maslahat*, ‘expediency’, interpreted in a secular encounter between Shi^c^a theological exegesis and modern statecraft. Secondly, crisis has not led to the production of a ‘state of exception’ as Agamben argues. Instead, since the late 1980s, a *sui generis* institution, the Expediency Council, has presided and decided over matters of crisis. Instead of leaving blind spots in the production of legislative power, the Expediency Council takes charge of those spheres of ambiguity where the ‘normal’ – and normative – means of the law would have otherwise failed to deliver. This is a first study of this peculiar institution, which invites further engagement with political phenomena through the deconstruction and theorization of crisis politics.

The *State secret* is truly the secret of a temple: the profanes shall not move close to it.

Carlo Levi, *Paura della libertà* [Fear of Freedom].^1^

In November 2011, the President of the European Union (EU), Manuel Barroso, quoted the following: ‘Europe will be forged in crises, and will be the sum of the solutions adopted for those crises,’ adding, ‘This still holds true today. Although the truth is also that today we are probably facing the *most serious* crisis in the history of European integration’.^2^ This statement falls along a long series of crisis announcements which surfaced more markedly in the aftermath of the economic crisis of 2008. Scholars interested in contemporary politics, whether political scientists, sociologists or anthropologists, have dedicated plenty to understanding the ways ‘crisis’ works in this epoch.^3^ Being natural disasters (earthquakes, droughts, floods), political turmoil (electoral impasse, electoral meddling), financial meltdowns (EU in 2008, 2013) or medical emergencies (HIV, bird flu (H5N1), mental illnesses), the category of crisis has come to occupy an eminent place in social science discussions and in matters of governance.

Terrorism, starting from the events of September 2001 in the United States, was instrumental in the framing of governance as being challenged by impeding crisis, which situates states and their governments in emergencies. Between 2015 and 2017, the French government extended multiple times the ‘state of emergency’ (hinting at its use also amidst popular protests (such as the *Gilets Jaunes* in 2018), an event that has no precedent since the Second World War. Indeed, it had no precedents even when one looks at the historical window of terrorist attacks during the 1970s and 1980s, for instance in the operations carried out by Ilich Ramìrez Sanchez, also known as Carlos the Jackal. Nonetheless, crisis operates beyond immediate concerns over security as in the case of terrorism. It applies to the framing and understanding of migration, economics (debt and austerity) or the environment (climate change, water shortages) and other fields of everyday governance. For instance, the Italian government declared a state of emergency in the wake of the 2016 earthquake that hit its central regions of Marche, Abruzzo and Umbria. This enabled the government to request an ease on the EU budgetary restriction and access extra-budgetary monies. Similarly, Italy has on several occasions stressed that it should be given an exceptional status, as it faces a refugee crisis, again permitting the unlocking of financial resources otherwise unavailable. The justification, in both cases, came under the necessity to face the *crisis*, which could not be dealt with by normal means and which, therefore, enabled governments to play beyond the agreed rules and restrictions of the EU. Lastly, Donald J. Trump’s use of ‘emergency funds’ to bypass legislative obstacles against his 2016 electoral pledge of building the wall along the US-Mexico border confirms that the notion of crisis stands now at the centre stage of governance.

The intensification of a discourse of crisis proceeds over the blurring of boundaries between what is legitimately policy (and the state) and what is performed at the level of political intervention. As Janet Roitman argues, the reference in modern politics to crisis is a blind spot, which is claimed constantly in the political rhetoric, but ‘remains a latency’.^4^ Paradigmatic of this argument are the crises of contemporary society: the crisis of capitalism, of culture, of politics, of finance, banking, antibiotics, welfare, democracy, terrorism, and illicit drugs and addiction. In this way, it is not so much important whether one can effectively identify what crisis is; rather, the announcement of crisis by statutory machines of power or their assemblages – for example public institutions, think tanks, experts, international agencies – and/or independent popular representatives is a sufficient condition for experiencing the crisis effect. In other words, there is a crisis when someone cries ‘crisis…’.

Hence, crisis occurs in between discursive and material lines. It is partly instrumental in the justification of so-called exceptional policies and partly a consequence of substantial events that changed the conditions under which states govern. In this article, these two aspects of crisis politics are considered in their own ambiguities, as crisis itself remains a fluid category which exists empirically and, at the same time, is invented. Crisis, used as both idiom and praxis, has become a central political and policy category in the global twenty-first century.

The aim of this article is to recognize this global trend in one of its least integrated ecologies, that of the Islamic Republic of Iran. Iran, in fact, is no exception to this rule of global politics. *The Economist* argued that ‘one reason for the Islamic Republic’s durability against what many would regard as overwhelming odds is the dogged but subtle *crisis management* of Ayatollah Ali Khamenei’.^5^ Yet, how could the management of crisis be the domain of one man, be it even the Supreme Leader? While crises are addressed collectively and politically in so-called liberal democracies, they are expected to be managed by single individuals outside the West.

The argument in this article runs against this type of framing. First, the article introduces what the place of crisis in the politics of Iran is. It historicises the way this notion has come to play a substantial role in the formation and transformation of the Iranian state. Then, it discusses how crisis has enabled the surfacing of specific practices and institutions charged with crisis management. In light of this, the article analyses how crisis has become an idiom of reform and of management in the Islamic Republic after the Iran–Iraq War (1980–1988), bearing in mind that politics globally has progressively adopted similar processes of formation over this period. The argument contributes to the deconstruction of leadership dynamics in Iran, moving beyond the oft-cited politics of personalities (e.g. Khamenei versus Rafsanjani; reformists versus conservatives) towards a study of political practice. Inspired by the work of Italian philosopher Giorgio Agamben, the article moves also beyond his conceptualisation of ‘state of exception’, which has been a recurrent theory in critical studies within the social sciences. Discussed in relation to developments in Western democratic rule, the state exception is a paradigm that, according to Agamben, engenders ‘zones of indistinction’ where the law does not apply, leaving humans in a condition of ‘bare life’.^6^ The state of exception is interpreted as a form of state impunity, a form of power where law and legal regulation do not apply, and which produce the non-juridical category of ‘crisis’ or ‘emergency’. In the coming pages, I revisit this claim in the context of the Islamic Republic of Iran, a country generally regarded as authoritarian and at odds with Western polities, one where arbitrary power should link to the state of exception and the non-juridical use of ‘crisis’. Contrary to Agamben’s postulate, in the Islamic Republic of Iran the category of crisis has been given, first, a juridical status through the institution of *maslahat*, ‘expediency’, interpreted in a secular encounter between Shi^c^a theological exegesis and modern statecraft. Second, crisis has not led to the production of a ‘state of exception’ as Agamben argues. Instead, since the late 1980s, a *sui generis* institution at the heart of the Iranian state, functions as its foremost crisis management machine: the Council for the Discernment of the Expediency of the State (*majma‘-e tashkhish-e maslahat-e nezam*, henceforth Expediency Council or the Council). It is the Expediency Council that has presided over matters of crisis.

This line of inquiry informs the argument in the direction of two conclusions: first, the zones of indistinction – and the state of exception – that qualifies the authoritarian model of states cannot be simplistically ascribed to the Iranian state since the Islamic Republic has produced juridical forms of governing the state of exception (thus, partly running against the grain of Giorgio Agamben’s theory). Second, the article concludes that crisis politics has had counterintuitive outcomes: the Islamic Republic has gone through a process of secularisation – largely because of its crisis politics – which resulted in a form of profane politics with oxymoronic outcomes, as I have elucidated elsewhere.^7^ In the latter part of the article, to give empirical face to the study of crisis management, the article looks at how the Iranian state has dealt with one of its long-standing crises, that of drugs and addiction.

The article is based on research fieldwork on crisis politics, drugs and state formation carried out inside Iran between 2012 and 2016. The article is based on a large database of government documents, public records, official decrees, publications by ministries and state agencies (Drug Control Headquarters, Expediency Council, several ministries) and commentaries on the Expediency Council accessed at the National Archive in Tehran. This has been further complemented by a dozen interviews with key members of the Council’s bureaucratic apparatus, members of its policy formulation body, non-governmental organisation (NGO) workers and medical experts, academics and representatives of international organisations such as the United Nations Office of Drugs and Crime (UNODC) and the World Health Organisation (WHO). Newspaper articles in Persian and reports published by other state institutions which discuss the role of the Council have also been considered. As one of the most obscure fields of contemporary politics of Iran, the article is of high relevance to all those interested Iranian politics, Islamic political development as well as for those studying state formation, crisis politics and comparative politics of liberal/illiberal states. Ultimately the article invites further reflection upon the possibilities for integration in the study of global politics beyond exceptionalist frames.

## Crisis effect in Iran

Crisis, its Greek etymology reveals, takes effect in the domains of theology, medicine and the law.^8^ From ancient to modern history, the word was equally used by religious ministers, physicians and jurists to refer to the ‘decision’ (*krisis*) upon life. For instance, there is a crisis when a doctor needs to take urgent decisions on a patient’s condition (the life-threatening situation), what we would call ‘emergency’; or in eschatological terms, on the Day of Judgement when decision upon eternal life is made; or in judicial terms, such as the state of emergency, when the norm is suspended. The Persian equivalent is *bohran* (of Arabic origin), which maintains the triple sense of the word ‘crisis’. These three dimensions converge when one looks at the state formation of the Islamic Republic. The political order brought into existence in 1979 combines public law with religious exegesis – there exists the *Islamic res publica* – and bestows upon medicine ample space for justification of policy intervention. Epidemics, diseases and the threat to public health become moments for the reconfiguration of public order and the state. Medicine becomes a primary device for the declaration of crisis as discussed below.

‘Crisis’ is not an exceptional condition in post-revolutionary Iran. In fact, one could argue that crisis has been perpetual ever since the establishment of the modernising state in the early twentieth century, witnessing a steep intensification in the aftermath of the 1979 Revolution. With the establishment of a revolutionary order, the public discourse on politics has been reproduced – not only by Iranian officials, but also among international observers – through recurrent allusion to the notion of crisis: ‘the US hostage crisis’, ‘the oil crisis’, ‘the war crisis’, ‘the post-war crisis’ of the 1990s, ‘the water crisis’ of the 2010s, ‘the corruption crisis’…and, ultimately, ‘the nuclear crisis’. Two examples from the last two decades hint at the discursive and substantial dimension of crisis politics: in the early 2000s, President Mohammad Khatami (1997–2005) declared that his government could not push for reforms because he was facing ‘a crisis every nine days of government’.^9^ His successor, Mahmud Ahmadinejad (2005–2013) presided over a government that was described by commentators, picking up on Khatami’s words, as one ‘facing nine crises every day of government’.^10^

The crisis that has had a long-lasting presence in both language and practice in the politics of Iran has been ‘the drug and addiction crisis’. Over the course of the twentieth century and, with increasing drama, following 1979, public discourse referenced the emergency of opium, heroin, and since the 2000s, methamphetamine consumption. With one of the world’s largest drug-using populations, Iran has historically been struggling with a drug problem.^11^ A first instance was given when the Society against Opium and Alcohol, created by a group of upper-class men and their wives at the end of the Second World War with the aim of eradicating opium and alcohol consumption, began publishing leaflets with astonishing numbers about the damage of substance abuse. Death, suicide, poverty, abandonment, unproductivity and immorality all worked towards the making of a crisis. The crisis was later picked up by policy-makers and used as proof of the need to introduce a prohibitionist bill on opium in 1955, which was justified on health grounds.^12^

After the Islamic Revolution in 1979, the new political cadre announced that the purification of the political body had to go through the cleansing of drugs from the public space. The question of drugs was so crucial to the new Islamist regime that it was put second on the list only after the Iran–Iraq War.^13^ Later towards the end of the 1990s, the outbreak of an HIV epidemic brought to light another dimension of the drug phenomenon. This time the crisis did not trigger further punishment and securitisation; instead, it enabled reforms. The HIV crisis, in the form of an epidemic, legitimised the adoption of harm reduction measures, a set of controversial policies that instead of criminalising drug users intended to provide them with basic medical and welfare needs.^14^ These included the provision of clean needles and injecting paraphernalia to heroin users, condoms to sex workers using drugs, methadone substitution treatment to opiate consumers and a shift in the public rhetoric vis-à-vis drugs. These practices were implemented also in prisons, therefore forcing the authorities to acknowledge that drugs were widely available even within an allegedly secure environment and implying the failure of the security approach towards the drug war. One should bear in mind that harm reduction approaches are opposed or restrained in a great number of Western liberal democracies, including the US, the UK and several EU countries. The drug crisis enabled responses that were previously unthinkable in the austere political environment of the post-war Iran (1990s). Calls for drastic reforms, changes in policy directions and imminent social and medical catastrophes have since then become common tropes. On this ground, I take the drug crisis as an analytical case for the study of crisis management. Its unwrapping is both discursive and substantial; crisis operates at the level of make-believe and, simultaneously, in effective terms. Hence, crisis is an idiom – an image and a language – as much as it is a lived condition and a praxis. In the context of post-revolutionary Iran, an institutional venue – a legal framework for (en)framing, announcing and managing crisis – contains the facet of crisis politics: the Expediency Council, to which the next section is dedicated.

## Genealogy of crisis politics and expediency

Crisis in Iran is handled in a legal venue; it is the Expediency Council, an institution made up of the leading political figures of the Islamic Republic, with a multi-layered bureaucracy and pluralistic connections to both the leadership, the ministries, the scientific community and civil society. The case of drugs policy is of especial importance to this institution, which performs at the same time executive, legislative and consultative tasks. In fact, all Iranian legislation is debated and, initially, formulated in the parliament (*Majles*), except for drug laws that are debated and formulated only in the Expediency Council. From a political standpoint, this has contributed to making the ‘drug problem’ into a permanent crisis for the Islamic Republic, especially since the end of the Iran–Iraq War in 1988. But before addressing this specific case study, I shall provide a brief genealogy of this *sui generis* institution.

In the words of Ayatollah Khomeini, the Expediency Council was expected to intervene in situations that ‘could not be solved through *normal* means’.^15^ Not much has been said about the Expediency Council in the academic literature. Generally, reference to this institution is limited to a few lines, or a paragraph, detailing its birth in the late 1980s and its role as mediator between the Parliament and the Guardian Council, a body overseeing legislative proposals disposed by the parliament and acting similarly to a standing Constitutional Court. Nonetheless, in the hierarchy of the Islamic Republic, the Expediency Council stands at the very top of the political machinery, in symbiotic relation to the Office of the Supreme Leader (*daftar-e maqam-e mo’azzam-e rahbari*) and has affected processes of state formation at fundamental historical junctures.^16^

The origin of this institution is opaque. Asghar Schirazi holds that, in practice, the Expediency Council had existed since 1981 as ‘an authority that can go over the head of the official government and decide on the most important questions of policy’.^17^ Its *modus operandi*, behind the scenes, may have paralleled that of other unelected councils with legislative power in the early 1980s, such as the Supreme Council of the Cultural Revolution and the Supreme Council supporting the War and that of Reconstruction. If this is the case – plausible given the practice of holding informal high-ranking meetings outside government venues – the Council started its activities coterminous with the critical period of state formation after the revolution and in concomitance with the war (1980–1988). One could interpret it as the materialisation of state prerogatives amid the multiple moral and political constraints of the early 1980s.

It is worth noting that up to the end of the war in 1988, the state-making approach accentuated, haphazardly, the notion of ‘rule of emergency’ in order to circumvent religious impediments. Based on the Koranic assumption that ‘emergencies make it permissible to do what is forbidden’, the Iranian state resorted, on several occasions, to this loophole (*escamotage*) to bring forth crucial political projects. The rulings approved by the parliament through this process were considered *zarurat*, ‘necessity’, and implemented as an experiment without going through the vetting process of the Guardian Council.^18^ Yet, the use of emergency as a device of governance lacked institutional venues and it addressed mostly the demands, expectations and social vibrations following the Revolution and the war efforts.

Over the 1980s, laws and bills approved by Parliament were often vetted by the Guardian Council, which in the Iranian political order plays the role of an unelected upper chamber (made up of six clerics appointed by the Supreme Leader in tandem with six civilian jurists selected by parliament). The Guardian Council vetted most of parliament’s laws on the ground that they clashed with the prescription of religious laws. The result was a stalemate between the Guardian Council and the Parliament which brought the legislative process to a standstill; the first was a conservative body, the other radical in its push for social reforms. The three branches of the state – Parliament, Judiciary and Government – eventually sent a letter to the highest authority of the state, Ayatollah Khomeini, requesting further clarification on how to enact a governmental ordinance that could speed up the political process. Khomeini, after having upheld that ‘government […] is one of the principal rules [*ahkam*] of Islam and it stands above all other rulings including prayer, fasting and hajj [pilgrimage]’,^19^ responded prescribing the establishment of the Expediency Council. The letter concludes with these lines:

…bear in mind that the interest of the political order [*nezam*] is among the important issues that, if ignored, can cause the failure of our dear Islam. Today the world of Islam considers the Islamic Republic of Iran a universal sign for the solution of its problems. The interest [*maslahat*] of the state and the people [*mardom*] is a fundamental issue that if opposed […] might give way to the American Islam of the arrogant and powerful with all the billions from within and without.^20^

Khomeini claimed that governance – the act/duty of governing – was the core element of Islam.^21^ In acknowledging the centrality of political imperatives – as opposed to religious ones – to the act of governing – even (or especially) in an *Islamic* state – implied, as reformist intellectual Saeed Hajjarian holds, acknowledging ‘the dynamicity of religious thought in its applicability with the requirements of the historical era and to the solution of problems and insufficiencies of society’.^22^ One could add that there is an apparent oxymoronic value in Khomeini’s above-mentioned statement: in order to save Islam from the danger of the secular world (the *American Islam*), the Islamic Republic needs to think outside Islam. It needs to become profane – ‘pro’ (in front) and ‘fanum’ (temple): *to stand out of the temple (of religion)* – in the form of a secular Islam. I shall bring back this consideration in the conclusion to this article.

The constitutional changes that took place at the end of the 1980s exemplify the political transformation occurring at the heart of the Islamic Republic. On 24 April 1989, 40 days before his death, Khomeini sent a letter to the then-president of the Republic Ali Khamenei and requested the creation of a Council for the Revision of the Constitution (*shura-ye baznegari qanun-e asasi*). Two issues were addressed in Khomeini’s decree: the issue of leadership; and the constitutional recognition of the Expediency Council of the State, the latter having been created in 1986 as a temporary institution to solve the stalemate between the Parliament and the Guardian Council.^23^

The revisions of the Constitution included cosmetic/ideological changes, such as the re-labelling of the Parliament from *majles-e shura-ye melli* (national council) to *majles-e shura-ye eslami* (Islamic council), to more structural amendments, such as the abolition of the post of Prime Minister and the transfer of the latter’s duties to the presidency. In addition, the Supreme Judiciary Council, which was tasked with all matters related to justice, was substituted with the Head of the Judiciary, directly appointed by the Supreme Leader. The highest political authority in the Islamic Republic remained the Supreme Leader, who just before Khomeini’s death had seen its office strengthened with new powers, upgrading it to the *velayat-e motlaq-e faqih*, ‘absolute guardianship of the jurist’. This new attribute allowed the leadership to issue ‘governmental ordinances’ (*ahkam-e hokumati*) when the political order (*nezam*)^24^ experienced instability, crisis or disorder. The ordinances could not be vetoed by parliament or the Guardian Council.^25^

Since the Parliament could not legislate outside the remit of the Constitution *and* of the official religion (i.e. Islamic law as interpreted by the Guardian Council), the governmental ordinances were meant to address those situations in which standard political intervention was problematic. The ordinances were based on two key elements: ‘the *ijtihad-e mostamerr* (permanent interpretative effort of the Islamic jurist), expected to update its interpretation of religious laws according to the changing of times’; and ‘the acknowledgment of advanced sciences [^c^ *olum*], arts [*fonun*] and experiences [*tajarob*] of mankind and their effort towards progress’.^26^ In other words, the governmental power brought in by the constitutional revision institutionalised the short-term political expediency that had characterised the management of the war and its politics of crisis, to which the creation and institutionalisation of the Expediency Council is the most paradigmatic response. It is an attempt to provide an institutional venue for crisis politics – a form of institutional exception.

If one pays heed to the Articles of the Constitution as revised in 1988, references to the Expediency Council occur in sections in which a situation of urgency or crisis is contemplated. Article 112 legitimises its establishment and defines its main duties:

Upon order of the Supreme Leader, the Expediency Council shall meet at any time the Guardian Council judges a bill proposed by the Majles to be against the principles of *shari^c^ah* [religious law] or the Constitution […]. Also the Council should meet for consideration on any issue forwarded to it by the Supreme Leader….^27^

Article 110 lists the responsibilities of the Supreme Leader, among which stands out ‘the resolution of the system’s problems which are not solvable in a normal way (*az tariq-e ^c^adi*), through the Council for the Discernment of the Expediency of the State’.^28^ This article enshrined the primacy of political reason in matters of statecraft and policy-making, the implication being that Islam *cannot* alone be the solution to all problems. To buttress the argument that this institution operates in situations of crisis and contingency, Article 111 establishes that,

If the leader is incapable of governing […] a council is formed with the president, head of judiciary, and one of the jurists of the Guardian Council as chosen by the Expediency Council. […] If for any reason one of the members of this temporary council cannot fulfil his role, the Expediency Council will appoint another in his place, maintaining the majority of clerics in the council.^29^

Again, it is the Council that decides [*κρíνω*] in situations of emergency. Finally, Article 177 allows revision of the Constitution only when the Supreme Leader, after consultation with the Expediency Council, indicates which parts of the text need to be amended.^30^ With the enshrining of this institution within the structure of the Islamic Republic, the state acquired the capacity to intervene in spheres that were religiously controversial. More importantly, Khomeini did not bestow the Supreme Leader (*velayat-e faqih*) (and religious law) with the ultimate power to rule over all matters of urgency that regard the state. It had made the state itself the ultimate authority with regard to all matters. Inevitably, this epochal transformation triggered criticism and a debate around the legitimacy of this paradigm of government.^31^ I shall now dwell on how the Expediency Council works.

## ‘Gazing eye, thoughtful brain’: structure, agency and power

The Expediency Council can be described as the state in a nutshell. It comprises the leading figures of the political establishment from all branches of the state. Every member is directly appointed by the Supreme Leader and holds a post for a renewable five-year term. There has been continuity in the membership of the Council, with a progressive increase of its size, although members who fell out with the political order (e.g. Mir-Hossein Musavi) have not seen their posts renewed. Presidents of the republic have regularly been appointed to the Council, as well as the Heads of Judiciary, members of the Guardian Council, influential Revolutionary Guards (IRGC) commanders, Directors of the Supreme National Security Council as well as Speakers of Parliament.^32^ Inevitably, the Council has been dominated by clerical elements, although laymen have seen their numbers on the rise.^33^ Read against the grain of its principal task – the interest of the state – the presence of clerics may have contributed to a further secularisation of their political attitudes when faced with political contingency of the profane type.

On 20 February 1997, the head of state Ali Khamenei issued a decree in which he outlined the new duties of the Expediency Council, adding to the constitutional duties, ‘the powers to determine the general policies of the state and major questions of the country; tackling of important issues on request of the Leadership as well as advising the Leadership’.^34^ Since then, the Expediency Council has operated as a Leadership Headquarters – beside the official Leadership Headquarters (*setad-e rahbari*) which is Khamenei’s office – on all political matters. In the words of its general secretary Mohsen Rezaei, ‘the Leadership needs an expert institution *with a gazing eye* and *a thoughtful brain* [emphasis added]’,^35^ and the Council has been unavoidably regarded as the only institution capable of operating as such (Table 1).^36^

**Table 1.** Structure of the Expediency Council up to 2017.
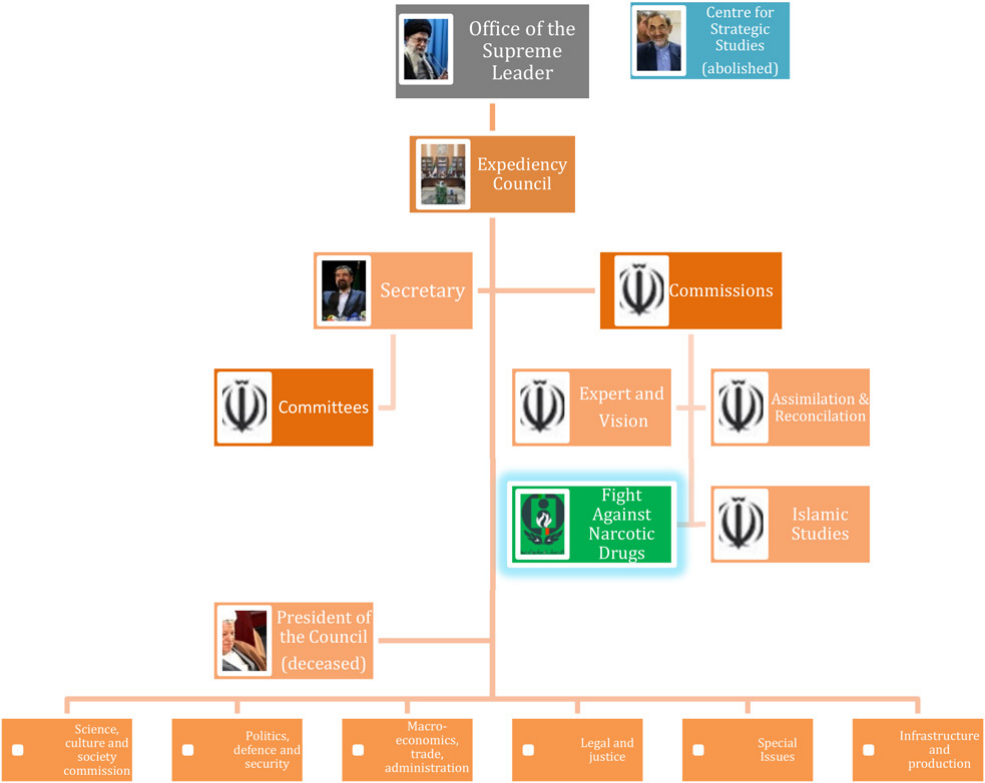

The Council adopts a secular structure divided into: presidency (Hashemi Rafsanjani: 1989–2017, Hashemi-Shahrudi: 2017–2018, Larijani: 2019–); secretary (Mohsen Rezaei: 1997–) under which operate several specialised commissions; six permanent commissions; and, up to summer 2017, the Centre of Strategic Studies acting as a research provider to the council.^37^ Each permanent commission has a chairman, a deputy and a secretary. The permanent commissions are expected to examine bills and proposals from the specialised commissions. They are divided according to the following fields: (1) Science, culture and society commission; (2) Politics, defence and security; (3) Infrastructure and production; (4) Macroeconomics, trade and administration; (5) Legal and justice; (6) Special Issues.

Four specialised committees operate instead under the Secretariat of the Council: (1) Experts and Visions/Prospects (*cheshmandaz*); (2) Assimilation/Reconciliation (*talfiq*); (3) Fight against Smuggling and Narcotic Drugs; (4) Islamic Studies.^38^ Overall, the only independent specialised committee that deals with a specific social and political urgency within the Expediency Council is the Drugs Policy Commission; but that does not imply that the Council does not intervene in other fields deemed urgent (e.g. land policies, media, death penalty, international treaties, financial regulation, state administration planning). Beside the above-mentioned duty to solve those problems of the Islamic political order that cannot otherwise find a ‘normal’ solution, the council performs its multiple duties according to an internal regulation which stresses, repeatedly, the use of ‘the most updated findings of expertise’, ‘the use of practical, developmental and foundational researches within the country’s research centres’, and ‘the use of experts from public and private sectors’ in the determination of its decisions.^39^

This aspect of the policy-making process through the Expediency Council has been highlighted by Khamenei himself. After the end of the war and the death of Khomeini, Khamenei revealed, the leadership sought to make use of the Council as ‘a collection of thoughts, interpretative efforts [*ejtehad*], expertise [*karshenasi*], experiences and adherence to the traditions and observance of the interest [*maslahat*]’. The Leader then concluded saying ‘I invite you to go beyond *factions* in the meeting of the Council. Here the question is the interest of the country.’^40^ If one goes beyond the rhetorical aspects of this message, the lack of any direct or indirect reference (apart from an opening eulogy) to Islam and, for that matter, religion is emblematic of the mechanisms embodied in this institutional body. Operating as a profane venue of confrontation among long-term political figures of the Islamic Republic, the Council also enjoys especial powers in terms of policy-making, beyond that of resolving conflicts between the Guardian Council and the Parliament.

Between the year of its establishment and its formalisation in the Constitution, the Expediency Council was authorised to pass laws without mediation from other state institutions, including the main legislative body of the country, the Parliament.^41^ Thus, the Council entrusted itself with fundamental legislative powers, as an extension to the Supreme Leader’s authority to solve problems *unconventionally*. The body as such had potentially far-reaching powers, which however in political practice have been exploited only in times of policy bottleneck, urgency and crisis. Neither the Parliament nor the Guardian Council can modify laws approved by the Expediency Council. Once a law is approved by the Council, the only procedure through which it can be updated, cancelled or reformed is another deliberation of the Council itself. The Council cannot be audited and investigated by other state institutions without the Leader’s consent, a fact that represents a strong exception given Iran’s parliament *de jure* comprehensive auditing power.^42^ The Council being unelected, these issues hint at a fundamental democratic and republican deficit, which gains momentum in times of political crisis and highlights the negligible checks and balances in the structure. But how does the actual process of policy formulation work within the Expediency Council?

On receipt of a request for intervention, whether by the Leader or by the Majles (in case of stalemate), the presidency of the Council refers the dispute or question to the secretary, who then introduces the matter to one of the relevant specialised commissions (under the Secretariat). The latter investigates the request in collaboration with the specialised independent committees (such as the Drugs Policy Committee), which expresses its opinion after evaluation and assessment by inviting experts on the issue; it then sends back the issue to the permanent commission of the Council. The latter evaluates it and, if it deems the proposal relevant and solid, the question is sent to the Council’s assembly for a final vote (Table 2).^43^ The deliberations of the Council are on a nominal majority vote (except when instructed otherwise by the Leadership, as in the case of the Palermo Convention vote in 2019) and need the endorsement of the Supreme Leader, a procedure that has hitherto been largely a formality.

**Table 2.** Structure of drug policy commission.
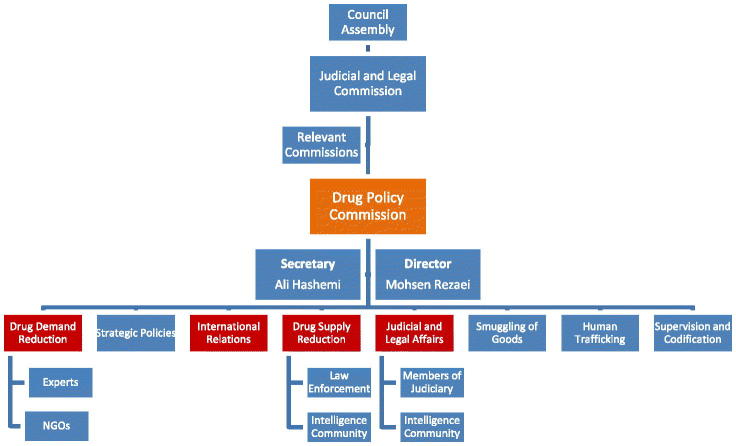

The Expediency Council, indeed, is the arena of confrontation at the core of the Islamic Republic. Each step of the evaluation happens with the participation of officials and the personnel of ministries relevant to the topic under debate, who can provide their input even in the final vote of the Council.^44^ For example, if the debate concerns public health risks, officials from the ministries of Public Health, the Welfare Organisation, the Prison Authority, representative of NGOs, as well as senior epidemiologists from universities and research centres would take part in the debate. In this, the Council is characterised by a certain flexibility in terms of its structure, membership and content of debate.

This fluidity and breadth of intervention has caused, as mentioned earlier, disapproval by members of the executive. For instance, President Hassan Rouhani criticised the intromission of the Expediency Council, in which he has been a long-lasting member, on the drafting of the 6th Development Plan which he argued was a priority of his government.^45^ Similarly, the Parliament has repeatedly expressed concerns over its incapability of legislating in areas in which the Council has already intervened, because the laws approved by the Council are unchangeable by other legislative branches.^46^ Accused of having become a sort of upper house, a Senate – which in Iran’s political parlance is inherently pejorative and illegitimate as it recalls the Pahlavi era (pre-1979) – the ambiguity of the Expediency Council (and its General Policies) within the Iranian system are regularly catechised. In 2013, the Supreme Leader delegated some of his powers of supervision to a sub-group of the Council, named the Supreme Group of Supervision which was responsible for harmonising legislative policies with General Policies of the state.^47^

One sector in which the Council has been permanently active and in charge is that of drug laws and drugs policy. I shall now turn to the Council’s management of the drug crisis.

## The expediency council on drugs policy

Between 1988 and 2001, the Expediency Council intervened in eight different circumstances on drug laws, marking drug legislation as the exclusive turf of this institution. While the Council has legislated on a wide spectrum of issues over the course of the last three decades, law-making has been ordinarily and legitimately carried out by the Parliament. For drug laws, in contrast, this exception has effectively been the rule. Khomeini spelled out clearly towards the end of the 1980s that ‘after the war, the most important question for the Islamic Republic is the problem of drugs’.^48^ With the end of the war, state-making efforts and social intervention shifted towards the other ‘imposed war’, that of drugs.^49^ The social phenomenon of drugs, also in view of the way it had been framed over the years following the Islamic Revolution, embodied a fundamental, political crisis. Instead of undertaking a standard legislative path through the parliament, which perhaps could have engendered mutual accusations of corruption, laxity, hypocrisy and anti-revolutionary behaviour, the Council, headed by then to-be Leader Ali Khamenei, presided over the first comprehensive draft of drug laws. It is emblematic that a most profane, yet ethically problematic, issue such as drugs became a question of *raison d’état* or *maslahat*.

On 29 May 1988, the Council approved the first Anti-Narcotics Law. This became a milestone which determined the architecture of Iran’s strategy on drugs for the years to come. The text included initially 40 articles that systematically addressed issues of illicit drugs trafficking (i.e. opiates, cannabis), punishments and fines as well as measures of intervention for drug addicts. The text of the law did not produce a radical change in terms of measures against drug use and trafficking, but it materialised as a ‘security and social necessity’ for the state.^50^ The objective was not to overhaul the security-oriented, punitive approach that had come into being following the Revolution; instead, the major concern that characterised it was to systematise and enshrine within the country’s legislative process a state-led approach to drugs, one that during the 1980s had been characterised by great revolutionary zeal, but little systemic engagement.^51^ Even on this occasion, the intervention of the Council was meant to be temporary and the Anti-Narcotics Law was envisaged to have a validity of two years, intended to face what was regarded as the transitory drug crisis. Instead, six months after its approval, the Expediency Council abrogated the two-year validity and entrusted the execution of all drug-related matters to a newly created institution, the Drug Control Headquarters (DCHQ), established by Article 33 of the law.

As during the 1980s, drug crimes were judged by the Revolutionary Courts, together with crimes against the revolution, blasphemy, national security and the state. Among the features of the revolutionary court, there is the fact that its deliberations cannot be reassessed and revised. Inevitably, this has led over the years to harsher punishments, with a weak judgement process, even in the cases that lead to death sentences. Although this provision has never been uprooted in the national drug laws, over the 1990s and, especially in the 2000s, the Council introduced a number of revisions to the drug laws, with important changes regarding sanctions and welfare provisions. Because the laws approved by the Expediency Council can be revised *only* by the Council itself, the issue of drugs – and drugs policy – situated itself in a condition of permanent crisis. In other words, drugs became a question of especial political importance for the Islamic Republic, one with which elected bodies could not interfere directly, and where the highest echelons of the state needed strategic evaluation and inter-institutional compromise. This condition of ‘dead-end law making (*qanungozari-ye bon-bast*)’, as one of my interlocutors in the Council put it, re-produced *crisis* in order to allow for the re-assessment of previously sanctioned laws.

An interview with a long-term member of the Expediency Council’s bureaucratic machinery unveils this condition:

At times, a social question [*mo^c^zal-e ejtema^c^i*] is not so relevant to people up to when it is transformed into a social phenomenon [*padideh-ye ejtema^c^i*] which grows and grows. When its limits go further, it becomes a concern to everyone. […] Sometimes, the worse it gets [*kharab-tar besheh*], the more it is to our advantage because it gets to a point at which we have to take a decision and when *the situation looks, or is, critical* then we can actually make decisions that are innovative. At that point, in political terms, you can transform the threat into an opportunity.^52^

In 2004, the Council modified the status of the Committee on Drug Policy into a Commission, given additional tasks and duties. Since then, the Commission has been made up of four specialised committees dedicated to: drug supply reduction; drug demand reduction; strategic policies; and international and transnational relations.^53^ The Council’s meetings on drugs policy, too, have increased from one meeting every two to three months to meetings on a fortnightly basis.^54^ The commission for drugs policy is headed by former IRGC commander Mohsen Rezaei, the secretary/director is former DCHQ official Ali Hashemi who supervises the work of the four sub-committees, the most important ones being drug supply and drug demand reduction.^55^

Rezaei has been member of the Council since 1997, where he acts as Secretary General with duties of supervision of the various committees and commissions as well as referral of their findings to the president of the council during general discussions and voting. Appreciated as a military commander – his 2015 return to the IRGC was a clear sign of this – he made a good case for his pragmatism when in 1981 amid the confrontation against superior Iraqi military forces, he stated:

We should accept that we are involved in a great revolution in which all the world has created an alliance against us, so we can’t overcome the war issue only by praying, we ought to increase our military science and technological power.^56^

As a secretary of the Council, he has also given prominence to these same characteristics by coordinating the evaluation process in the lower committees with expert members of the research community in Iran. Known to be a pragmatist in the Western scholarly discourse, the interesting elements rest on the emphasis that Rezaei has put throughout his career on scientific and technological advancement, something that has been largely utilised in drugs policy approaches over the last two decades.^57^

His closest advisor in the Drugs Policy Commission is Ali Hashemi, formerly at the head of the DCHQ. Hashemi is a high-ranking bureaucrat who has acted over the course of the last decades as advisor to President Mohammad Khatami (1997–2005), member of the National Security Council (1986–1997) and member of the IRGC (1979–1986). His contribution to the field of drugs policy is guided by public health considerations and an understanding of national security inclusive of public health matters. Indeed, under the reformist presidency, he actively supported the expansion of humanitarian and reform-oriented policies on addiction, justifying it in terms of social security and risk minimisation, including in national security terms.^58^ He is the foremost representative of harm reduction strategies and humanitarian drugs policy in the political order.

Changes within drug laws are first discussed at the relevant committee level; for issues related to public health, such as prevention and drug (ab)use, that is the demand reduction section; for issues related to smuggling and trafficking, it is the supply reduction section. Once the matter is discussed in detail and matured, a proposal or agenda of discussion (*dastur-e kar*) for policy change is sent to the Expediency Council’s Judicial and Legal Commission, at the top of which sits the Head of the Judiciary. This step is usually considered the litmus test for any new policy proposal and it is the site of institutional vetoes and stubborn opposition. Here, the views, according to my interlocutors, are generally security-oriented and punitive with regard to drugs policy, although important changes have been taking place over the course of the last decade.

The participation of expert panels, practitioners, scholars and civil society groups is standard practice of the evaluation process. For issues related to drugs policy, well-known personalities from the medical community are generally invited to present on specific topics of interest to the sub-committees or the Council. Prominent epidemiologists within Iranian universities and leading members of NGOs provide their accounts and analyses at this level, with a focus on tangibility of results and experiences rather than political readings of the problematique. There is a general predilection for numerical reports and econometric results, which often facilitate the committees to make the case for or against a policy proposal. It is established practice to have the participation of experts known for their opposing views; although this could be complex to apply at all times, it is the norm in debates about drug law reform. Collaboration with research centres is instrumental to the policy debate within the Council. Evidence gathered from the ‘field’ of drugs policy is mediated by researchers and put at the disposal of the bureaucratic apparatus to be prepared for the Council’s debates.

One research centre that has played an important role in this is the Iranian National Center for Addiction Studies (INCAS). Created in 2004, amid the controversies around ‘harm reduction practices’, INCAS is a venue for research *and* implementation/design of drugs policy and, indeed, it played a central role in the frame of the pilot methadone programme, by providing a scientific language and policy evidence for its scaling up.^59^ Based on its status as an experimentation and research centre, INCAS has contributed to policy design in the field of addiction not only in Iran, but also in Afghanistan and Pakistan.^60^ Similarly, Iran’s counterintuitive programme for treatment of alcohol dependency was initiated within INCAS, then received final approval from the Ministry of Health on the advice of the Council.^61^ The use of such a medical research centre hints at the traditional acceptance of medical expediency in Shi’ite religious jurisprudence, where substances and/or actions deemed *haram* (forbidden) can generally be reputed licit if they are proved beneficial to the believer’s health.^62^ The clergy’s approach to birth control, research on stem-cells and the right to use narcotic drugs for medical purposes are examples of this medicalised rationale at the base of jurisprudential arguments and policy-making.^63^

Studies of the economic cost of drug (ab)use and the benefits of a reformed approach – such as decriminalisation of drugs – have also been submitted to the Council by independent researchers or affiliates to INCAS. For instance, several studies were provided in favour of harm reduction practices in reducing both costs and harms of drug (ab)use during the early 2000s. Among these, an influential comparative study was produced by Hooman Narenjiha and Roya Noori on the pilot experiment programmes in Kermanshah and Tehran, where it is argued that by introducing harm reduction, Iran could potentially save 400 billion human, equivalent to eight budgets of the DCHQ in 2006.^64^ The proposals and reports sent to the Council can be accepted, rejected or sent back for revision – similarly to an academic peer-review process, although the Council’s timescale might take fewer months. After this step, the proposal can be reviewed by other relevant commissions if it pertains to their field of intervention. The Expediency Council eventually puts the question to a vote. Given that members of the Council also sit on lower-level committees, proposals that arrive at the Council level tend to be approved without major impediments and are sent for final endorsement by the Supreme Leader. A first major instance of drugs policy reform was represented by the adoption and expansion of a ‘harm reduction’ policy (*kahesh asib*/*zayan*) on a nationwide scale. The adoption of these measures, beside their phenomenological rationale and sociological evaluation, casts light also on the practicalities of the policy-making process through which this institution formulates drugs policy. Inputs from NGO workers, medical researchers and international experts, coupled with the perception and materialisation of a crisis, facilitated re-formulation. This input occurred with an ‘economic orientation’ and a ‘capital calculation’ which informed the assessment of the Expediency Council, and which embody two distinctive rationales of this institution.^65^

The Council also promoted the inclusion of the harm reduction policy within the text of the Major Policies of the Islamic Republic in 2010, consolidating the legitimacy of this notion within Iran’s legislation (while the term remains taboo for the United Nations (UN) agency working on drugs and crime). Similarly, the 6th Development Plan, which for the first time was drafted by the Council, includes a reference to drugs policy in Article 22. This article states that the target of the Islamic Republic is, by the end of 2022, to reduce by 25 per cent the national rate of addiction. The article also adds that, in furtherance of this objective, the government should seek ‘the management of drug use in the country’, a statement that could be a prelude to shifts towards depenalisation and regulation of certain types of drugs.^66^ Unsurprisingly, there have been formal discussions within the Expediency Council, about ‘heroin shooting rooms’, regulation of opium and cannabis production, depenalisation of drug use and abolition of the death penalty for drug crimes.^67^ These debates are at times preceded by informal meetings in conference venues where leading members of the drugs policy community meet and discuss together with representatives of the Expediency Council.

From a legislative point of view, the Council’s strategy vis-à-vis drugs affect the rest of the political machinery; the Council has become the arena for confrontation and synthesis of different governmentalities regarding crisis. Reform of national policies is not driven by the ideological persuasion of different leaders or political networks, as most of the literature hitherto argued. Instead, political change and policy update is the outcome of multi-layered dialogue across institutions and knowledge centres. When it concerns critical matters, it is the Council that overtakes the framing, formulation and formation of policies. State formation, therefore, passes through the deliberations of the Expediency Council. In this political power resides within the logics of this institution more than with other specific agents that have traditionally been identified as ‘the regime’.

## Conclusion: who decides on crisis?

Crises operate in such a way that allows societal forces to push for change in certain fields, where governments have previously been unwilling or reluctant to intervene. But how does politics diagnose a crisis? And how are different social categories and social phenomena treated by political institutions when they are under (invented or material) conditions of crisis?

The Italian philosopher Giorgio Agamben argues that in contemporary governance the use of ‘emergency’ is no longer provisional, but ‘constitutes a permanent technology of government’,^68^ and has produced the non-juridical notion of crisis. It is the engendering of these zones of indistinction between the law and its practice, to which Agamben applies the notion of the *state of exception*. In the words of the author himself,

[the state of exception] defines a ‘state of the law’ in which, on the one hand, the norm is in force [*vige*] but is not applied (it has no ‘force’ [*forza*]) and, on the other, acts that do not have the value [*valore*] of law acquire its ‘force’.^69^

Conversely to this line of thought, in the Islamic Republic the category of crisis has been given, first, a juridical status through the institution of *maslahat*, ‘expediency’, interpreted in the secular encounter between Shi^c^a theological exegesis and modern statecraft. Second, crisis has not led to a formal production of a ‘state of exception’ as Agamben argues. Instead of leaving blind spots in the production of legislative power – and therefore the law – the Expediency Council takes charge of those spheres of ambiguity where the normal means of the law would have otherwise failed to deliver. This is no minor event, if one bears in mind that so-called illiberal states such as Iran are often recorded as producing states of exception beyond the realm of the law and of jurisprudence. The Islamic Republic falls outside the classical paradigm of illiberal, authoritarian state.

First, this prognosis of crisis is rooted in the ‘modern conceptualisation of politics and the political’;^70^ and because crisis operates as a narrative device regulating the framing of the present (or of history), it functions also as an analytical category, a prism of understanding of complex phenomena throughout historical progress. However, the prognosis of crisis is one moment within its management and does not imply that political practice is ridden with the mushrooming of states of exception, where people exist in non-juridical zones of ambiguity as Agamben argues.

Second, the management of crisis operates also through other apparatuses. These can be social service organisations, medical personnel, charity workers and volunteers.^71^ According to the definition, apparatus is a ‘device of population control and economic management composed of disparate elements that coalesce in particular historical conjectures, usually moments identified as “crises”’, composed of ‘discourses, institutions, architectural arrangements, policy decisions, laws, administrative measures, scientific statements, moral and philosophical propositions…’.^72^ Because the framing of the ‘drug problem’ has rhetorically and materially produced and reproduced multiple lines of crises – health, social, ethical – both globally and locally, an array of different and often apparently incoherent apparatuses have emerged. In Iran, the Expediency Council has emerged as a sovereign apparatus on the drug crisis – but decisively beyond the realm of narcotics too, as its expanding fields of intervention demonstrate.

And finally, historically regarded as a conservative institution in the scholarly literature,^73^ the Expediency Council has acquired positive power, in that it has enabled new regulatory situations and reforms even though most of its members are so-called conservatives. Rather than revealing a fundamentally regressive nature in relation to policy and polity at large, the debates within the Expediency Council are revelatory of an underlying secular logic governing the framing of critical phenomena. The Council’s sub-group overseeing parliamentary bills with the objective of fulfilling the General Policies of the State is a sign of its increasing centrality in governance and political vision, and a decreasing relevance of legalistic, jurisprudential bodies such as the Guardian Council. This produces what in the context of the Islamic Republic I call *profane politics with oxymoronic outcomes*. It is a form of politics that stands out of the temple of religion – even when its language and political habits are religiously disguised – and follows a logic driven by the challenges of the present – of the epoch (*seculum*) – rather than the timeless dimension of religious prophecies and revelations. The result is an oxymoron: the embracing of the dialectical and open-ended nature of politics as embedded in historical time.

In the case of contemporary Iran, this proverbial secularity is best captured in a statement made by a high-ranking official in one of the Expediency Council’s publications on the effects of subsidy reform on Iran’s drugs problem:

If one considers major drug traffickers and dealers, *from a Foucauldian analytical perspective*, their presence in the sphere of trafficking is motivated by the acquisition of a *power* which lies behind the veil of trafficking itself (as long as we see power as an expression of its three faces: capital, status and politics).^74^

The ‘normal’ limits that apply to politics and political rhetoric are defied within the Expediency Council, as the adoption of post-modernist ways of interpreting the world. The institutionalisation of crisis within the Islamic Republic’s governmental machinery is manifested in the establishment of the Expediency Council. Through the establishment of this political body, the Islamic Republic avoided the formation of multiple zones of indistinction, so typical of illiberal regimes (and increasingly of liberal ones).^75^

The Council stands at the highest core of legislative power, immune to the vicissitudes of electoral politics as well as unchained by the nuances of Islamic law: it intervenes in conditions judged – autonomously – exceptional and of crisis. Not only had this signature of power signified a reification of secular elements (meaning elements belonging to the time of the present) by the end of the 1980s, such as the inviolability of the state and primacy of political acumen in place of revolutionary and spiritual leadership. It also meant that, in terms of political praxis, the Expediency Council, not the Supreme Leader, has had ultimate governmental power in relation to crisis. And if sovereignty is defined by s/he who decides upon crisis, then it is time to paraphrase Foucault’s *bons mots*: the Supreme Leader reigns, but the Expediency Council governs.

## Notes

1 (Vicenza: Neri Pozza, 2018), p.54.

2 Jean Monnet quoted in Manuel Barroso, ‘A Europe for All Weathers’, Meeting with EU Heads of Delegation, Brussels, 30 November 2011, available at http://europa.eu/rapid/press-release_SPEECH-13-346_en.htm.

3 Vincanne Adams, *Markets of Sorrow, Labors of Faith: New Orleans in the Wake of Katrina* (Durham: Duke University Press, 2013); Didier Fassin and Daniela Pandolfi, *Contemporary States of Emergency: the Politics of Military and Humanitarian Interventions* (New York: Zone Books, 2010); Achille Mbembe and Janet Roitman, ‘Figures of the Subject in Times of Crisis’, *Public Culture* Vol.7, No.2 (2010), pp.323–52; Peter Redfield, *Life in Crisis: The Ethical Journey of Doctors without Borders* (Berkeley, CA: University of California Press, 2013); Janet Roitman, *Anti-Crisis* (Durham: Duke University Press, 2013).

4 J. Roitman, *Anti-Crisis*, p.39.

5 Emphasis added. *The Economist*, 5 May 2012, available at http://www.economist.com/node/21554242.

6 Giorgio Agamben, *Stato Di Eccezione* (Turin: Bollati Boringhieri, 2003), p.38.

7 See also Maziyar Ghiabi, *Drugs Politics*: *Managing Disorder in the Islamic Republic of Iran* (London: Cambridge University Press, 2019).

8 On the etymology of the word ‘crisis’, see Agamben’s interview, ‘The Endless Crisis as an Instrument of Power’, available at https://www.versobooks.com/blogs/1318-the-endless-crisis-as-an-instrument-of-power-in-conversation-with-giorgio-agamben.

9 Ghoncheh Tazmini, *Khatami’s Iran: The Islamic Republic and the Turbulent Path to Reform* (London: I.B. Tauris, 2009), p.127.

10
*Fararu*, 1 August 2009.

11
*Financial Times*, 2 January 2015, available at http://www.ft.com/cms/s/0/bcfb34ea-3e81-11e4-a620-00144feabdc0.html#axzz3VafleUT.

12 Maziyar Ghiabi, *The Opium of the State: Local and Global Drug Prohibition in Pahlavi Iran, 1941-1979* in Roham Alvandi (ed.), *The Age of Aryamehr*: *Late Pahlavi Iran and Its Global Entanglements* (London: Gingko Library Press, 2018), pp.70–109.

13 Maziyar Ghiabi, ‘Drugs and Revolution in Iran: Islamic Devotion, Revolutionary Zeal and Republican Means’, *Iranian Studies* Vol.48, No.2 (2015), pp.139–63.

14 See Maziyar Ghiabi, ‘Maintaining Disorder: The Micropolitics of Drugs Policy in Iran’, *Third World Quarterly* Vol.38, No.2 (2018), pp.277–297; for a narrative of the pre-2005 period, see Janne Bjerre Christensen, *Drugs, Deviancy and Democracy: The Interaction of State and Society* (London: I.B. Tauris, 2011).

15 Seyfollah Sarrami, *Ahkam-e Hokumati va Maslahat* [State decrees and expediency] (Tehran: ^c^abeir, Center of Strategic Studies, 1380 [2001]). Emphasis added.

16 The Office of the Supreme Leader is the bureau that coordinates, oversees, assesses and advises the operation of state institutions.

17 Asghar Schirazi, *The Constitution of Iran: Politics and the State in the Islamic Republic* (London: I.B. Tauris, 1997), p.95.

18
*Majmu^c^e Mosavabat-e Majma^c^e Tashkhis-e Maslahat-e Nezam* [Collection of Articles by the Expediency Council] (Tehran: Expediency Council, 1388 [2009]).

19
*Majmu^c^e Mosavabat*, p.13.

20 On 5 February 1988. *Majmu^c^e Mosavabat*, p.20.

21 For a theoretical discussion on this aspect, cf. Naser Ghobadzadeh, *Religious Secularity: A Theological Challenge to the Islamic State* (Oxford: Oxford University Press, 2014), p.27.

22 Saeed Hajjarian, *Jomhuriyat; Afsunzodayi Az Qodrat* [Republicanism: Demystification of Power] (Tehran: Tarh-e Nou, 1379 [2000]).

23 Said Amir Arjomand and Nathan J. Brown, *The Rule of Law, Islam, and Constitutional Politics in Egypt and Iran* (New York: SUNY Press, 2013), p.33. While Arjomand refers to this institution as ‘Maslahat Council’ and Schirazi calls it ‘Assessment Council’, I prefer to adopt the official jargon used by the Iranian authorities. In Persian, politicians and commentators usually refer to this institution simply as *majma’* (Council) or *maslahat-e nezam* (State Expediency).

24
*Nezam* is the Persian/Arabic word for ‘system’. Its etymological origin however hints at the Arabic root ‘nzm’, indicating ‘order’. As such, the word *nezam* could be interpreted as ‘political order’, ‘state’. In this article, I use the two words interchangeably.

25 Since 2014, the use of presidential ordinances has been on the rise, for instance, in the French Republic. This has occurred amidst stalemate and strong opposition against the labour law in 2018.

26 S. Sarrami, *Ahkam-e Hokumati.*

27
*Majmu^c^e Mosavabat*, pp.42–4.

28 Ibid., p.43.

29 Ibid., p.43.

30 Ibid., p.44.

31 For the diatribe, *Fars*, 30 November 2015, available http://www.farsnews.com/13940908001164.

32 The Supreme National Security Council (*shura-ye ^c^ali-yeh amniyat-e melli*) is an unelected body that oversees the top foreign policy and security issues in Iran. The Revolutionary Guards, also known as *pasdaran*, are a military force created in the wake of the Islamic Revolution in 1979 to prevent counterrevolutionary activities. They are today one of the two armed forces of the Islamic Republic of Iran.

33 It is no surprise that women have been excluded from the council’s assembly, since women have yet to occupy top offices such as the presidency, the judiciary, the speaker of parliament and other top posts in the political order. Female ministers, judges and vice-presidents have taken part in the meetings; female experts have also been invited to advise the Council on specific issues.

34 S. Hajjarian, *Jomhuriyat*, p.414.

35 Ibid., p.414.

36 With the sudden death of Ayatollah Hashemi-Rafsanjani, the Expediency Council underwent important changes in terms of composition. After the brief chairmanship of former Head of Judiciary Ayatollah Hashemi Shahroudi, who died in 2018, Ayatollah Ali Khamenei appointed Ayatollah Sadegh Ardeshir Larijani as the new head of the council. The table is made from information gathered on http://maslahat.ir/.

37 Since Hassan Rouhani’s election to the presidency in 2013, Khamenei appointed his long-term advisor on foreign policy, Ali Akbar Velayati as director of the Centre. In 2017, a reform of the Council put an end to the work of this centre.

38 Expediency Council, ‘Dabirkhane-ye *Majmu^c^e*’, available at http://maslahat.ir/DocLib2/Irec%20Pillars/Irec%20Secretariate.aspx.

39 Respectively, Articles 4 and 15 of the Internal Regulations of the Expediency Council.

40
*Majmu^c^e Mosavabat-e.*

41 Silvia Tellenbach, *Principle of Legality in the Iranian Constitutional and Criminal Law* in S. Arjomand and N. Brown (eds), *The Rule of Law* (New York: SUNY Press, 2013), pp.104–6.

42 Farideh Farhi, *Constitutionalism and Parliamentary Struggle for Relevance and Independence* in S. Arjomand and N. Brown (eds), *The Rule of Law* (New York: SUNY Press, 2013), pp.123–52.

43 Information gathered on http://maslahat.ir/.

44 Interview [public official from the Expediency Council], September 2015.

45
*Fars*, 20 December 2015, available http://www.farsnews.com/13940908001164.

46
*Aftab New*s (2014), available at http://aftabnews.ir/fa/news/243706.

47 See *ISNA*, ‘*Dar-bareyeh yek ta^c^vizeh ekhtiari az suy-e rahbar-e enqelab* [On an optional substitution by the Leader of the Revolution]’, available at https://www.isna.ir/news/94012408527.

48 Visible on posters and graffiti around the country.

49 The post-revolutionary rhetoric has referred to the Iran–Iraq War as an ‘imposed war’ (*jang-e tahmili*) by the West on Iran’s revolution. The War on Drugs, similarly, has been regarded as a ‘second imposed war’ on the Iranian nation. See M. Ghiabi, ‘Drugs and Revolution in Iran’.

50 Said Madani Qahrfarkhi, *E^c^tiyad Dar Iran* [Addiction in Iran] (Tehran: Nashr-e Sales, 1390 [2011]).

51 M. Ghiabi, ‘Drugs and Revolution in Iran’.

52 Interview (anonymous), September 2015.

53 Expediency Council, ‘Pishineh’, available at http://maslahat.ir/Commissions/AntiDrug/DocLib/History.aspx.

54 Interview with Said Sefatian, Tehran, September 2015. This Commission has been headed by Ali Hashemi, formerly Director of the DCHQ.

55 The DCHQ is the umbrella organisation overseeing all drug-related matters, in terms of implementation, research and evaluation. See www.dchq.ir.

56
*Al-Monitor* (2015), available at http://www.al-monitor.com/pulse/originals/2015/05/iran-mohsen-rezaei-irgc-return.html.

57 M. Ghiabi, ‘Maintaining Disorder’.

58 Interview with Said Sefatian, Tehran, March 2014. See Hashemi’s CV available at http://maslahat.ir/Commissions/AntiDrug/Documents.pdf.

59 Interview with Hamid-Reza Tahernokhost, UNODC expert on drug demand reduction, Tehran, July, 2012.

60 MENAHRA website, available at http://www.menahra.org/en/short-term-fundings/menahra-knowledge-hubs/incas/incas-about.

61 See INCAS website, available at http://incas.tums.ac.ir/page.php?slct_pg_id=309&sid=1&slc_lang=fa.

62 Maziyar Ghiabi et al., ‘Islam and Cannabis: Legalisation and Religious Debate in Iran’, *International Journal of Drug Policy* Vol.56 (2018), pp.121–7.

63 See for the *in vitro* fertilization, Morgan Clarke, ‘Children of the Revolution: ^c^Ali Khamene'i's “Liberal” Views on in vitro Fertilization’, *British Journal of Middle Eastern Studies* Vol.34, No.3 (2007), pp.287–303.

64 Interview with Hooman Narenjiha, March 2014.

65 Cf. Fariba Adelkhah, *Being Modern in Iran* (New York: Columbia University Press, 2001), p.166.

66 ‘Kahesh-e 25 dar sad-e E^c^tiyad dar Keshvar ta Payan-e Barnam-e Shishom-e Touse^c^’, Government of the Islamic Republic of Iran, available at http://www.dolat.ir/NSite/FullStory/News/?Serv=0&Id=266177. See also, M. Ghiabi et al., ‘Islam and Cannabis’.

67 Interview with Said Sefatian, 2015; see also *Sharq*, 10 September 2016.

68 Giorgio Agamben, *Stato Di Eccezione* (Turin: Bollati Boringhieri, 2003), p.38.

69 Ibid., p.38.

70 J. Roitman, *The Anti-Crisis*, p.22.

71 See Michel Foucault, ‘Il Faut Défendre La Société’. *Cours au Collège de France*, 1976 (1997).

72 Gregory Feldman, *Illuminating the Apparatus: Steps toward a Nonlocal Ethnography of Global Governance* in Chris Shore, Susan Wright, and Davide Però (eds), *Policy Worlds: Anthropology and the Analysis of Contemporary Power* (New York: Berghahn Books, 2011), p.32.

73 F. Khosrokhavar and M.A. Ghaneirad, ‘Iran's New Scientific Community’, *Iranian Studies* Vol.39, No.2 (2006), pp.253–67.

74 Expediency Council, ‘Asar-e Ejrai-ye Qanun-e Hadafmandi-ye Yaraneh-ha bar Eqtesad-e Mavadd-e Mokhadder’, Expediency Council, undated [2012?].

75 An example is given by the use of ‘courts of exception’ (*tribunaux d’exception*) in France, which deal with matters of terrorism and other extranormative issues, based on the fact that ‘the normal means of justice would have otherwise failed to deliver’. See Vanessa Codaccioni, *Justice d'exception. La cour de sûreté de l'Etat sous la Ve République: L'État face aux crimes politiques et terroristes* (Paris: CNRS, 2015).

